# A method for computing the overall statistical significance of a treatment effect among a group of genes

**DOI:** 10.1186/1471-2105-7-S2-S11

**Published:** 2006-09-26

**Authors:** Robert Delongchamp, Taewon Lee, Cruz Velasco

**Affiliations:** 1 Division of Biometry and Risk Assessment, National Center for Toxicological Research, Jefferson, Arkansas 72079 USA; 2 School of Public Health, LSU Health Science Center, New Orleans, LA 70112 USA

## Abstract

**Background:**

In studies that use DNA arrays to assess changes in gene expression, our goal is to evaluate the statistical significance of treatments on sets of genes. Genes can be grouped by a molecular function, a biological process, or a cellular component, e.g., gene ontology (GO) terms. The meaning of an affected GO group is often clearer than interpretations arising from a list of the statistically significant genes.

**Results:**

Computer simulations demonstrated that correlations among genes invalidate many statistical methods that are commonly used to assign significance to GO terms. Ignoring these correlations overstates the statistical significance. Meta-analysis methods for combining p-values were modified to adjust for correlation. One of these methods is elaborated in the context of a comparison between two treatments. The form of the correlation adjustment depends upon the alternative hypothesis.

**Conclusion:**

Reliable corrections for the effect of correlations among genes on the significance level of a GO term can be constructed for an alternative hypothesis where all transcripts in the GO term increase (decrease) in response to treatment. For general alternatives, which allow some transcripts to increase and others to decrease, the bias of naïve significance calculations can be greatly decreased although not eliminated.

## Introduction

The purpose of this work is to evaluate the statistical significance of treatments on the expressions in subsets of the genes on an array; for example, sets defined by gene ontology terms (GO terms, ). GO terms group genes according to a biological process, molecular function, or cellular component. Inferences about the impact of a treatment are usually more straightforward when based on GO terms or equivalent groupings as opposed to lists of significant genes. Hence, we want to assess the statistical significance of the treatments on the group.

mRNA levels measured among genes within a GO group will be correlated. Correlations among genes involved in a common biological task are likely, and correlations are also expected simply because the set of expressions are measured within an animal and array, i.e., under shared conditions. The p-values computed in many packages[1,2] assume independence and therefore could be misleading.

The statistical significance of a GO group is commonly assessed by counting the number of statistically significant genes in the group. The null hypothesis that this count is a random sample of the significant genes on the array is tested versus an alternative hypothesis that the count is enriched [1-3]. The test is Fisher's exact test (probabilities computed using a hypergeometric distribution) or one of its many approximations (e.g., chi-squared test which approximates the hypergeometric distribution with a binomial distribution) [4].

We do not test for enrichment primarily because the null distribution depends upon effects among interrogated genes that are unrelated to genes in the evaluated GO term. Put another way, the significance of a GO term should not depend upon whether or not other genes on the array (not in GO term) were affected by the treatments. This approach is not amenable to corrections for correlations among the p-values, since the test inherently assumes exchangeability among genes; an assumption which is not met under arbitrary correlation structures. Furthermore, the usual implementation simply counts 'significant' genes which precludes extracting supporting evidence from the 'not significant' genes.

The distribution of p-values for the individual genes on the array allows one to estimate the number of affected genes, and this estimate typically is larger than any list of 'significant' genes, which can be compiled with an acceptably low rate of misclassification [5-7]. Conceptually, methods that collate individual p-values within biologically meaningful groups can extract any supporting evidence for treatment effects from group members that individually cannot be identified as affected by the treatments.

We prefer an approach that is based on a p-value having a uniform distribution under the null hypothesis [8]. For any continuous probability distribution, *x *= *F*^-1^(1-*p*) transforms *p *to the distribution specified by *F*. Two choices for *F*, which are in common use for combing p-values, are the standard normal distribution, Φ(*x*), and the chi-square distribution with 2 degrees of freedom, *F*(*x) *= 1 - exp(-*x*/2). When p-values are independent, the distribution of the sum is straightforward for either normal or chi-squared deviates, and serves as a basis for testing the significance of a GO term. This is elaborated for the normal distribution in the next section, where we also develop a correction for correlations. The test based on the chi-squared deviate can also be corrected for correlation at least in a 'one-sided' case [9-12].

Randomization tests can deal with correlations among the genes in a GO term, and, when applicable, they should generate uniformly distributed null *p*-values [13-15]. Our studies usually process samples in batches and the estimation of treatment differences is essentially done within batches [6,16-18]. Such analyses usually cannot be implemented as randomization tests and our motivation to develop the presented methods is largely in this context. We will present adjustments for a one-sample t-test because the results are transferable to pair-wise contrasts in a fixed-effects linear model. While the pair-wise contrasts are our real concern, their presentation carries algebraic baggage that is largely irrelevant to correcting for correlation and we have opted for streamlined notation, which focused on the fundamental issues in correcting for correlation. A one-sample t-test can also be implemented as a randomization test, which suggests presenting it as a competitor. The randomization test is constructed to have a uniform distribution under the null although for sample sizes as small as *n *= 5 the number of possible permutations may be a complicating factor. When both methods are applicable, we would not choose between them based upon their abilities under the null distribution but rather on a consideration of their power and/or robustness, which is well beyond the scope of this paper.

We will assume that the data are *n *observations coming from a population with mean vector, **Δ **and covariance matrix, **Σ**. Although simple, this model is directly applicable to some of our studies, and it serves here to focus on basic issues with relatively simple mathematics. For an example where this model can be used directly, see the test for gender differences in gene expression as estimated in Delongchamp et al. (2005)[16].

## Methods

### Meta-analyses

Several methods are used in meta-analyses to combine a set of p-values into an overall significance level. Under a null hypothesis, the p-value for a corresponding statistic is a random variable with a uniform distribution and it can be transformed to a convenient probability distribution [8]. Here, we use the inverse of the standard normal distribution. Then

*z*_*i *_= Φ^-1^(1 - *p*_*i*_)

is a random variable from the standard normal distribution, and when the set of p-values, {*p*_*i *_: *i *= 1,...,*m*}, are also independent, the statistic,



where _*m*_**1**_1 _= (1,1,...,1)', also has a standard normal distribution. So, the p-value,



gives an overall significance level for the set. We refer to this as the naïve estimate because it naively assumes that covariance of **z **is the identity matrix, cov(**z**) = **I**.

### Adjusting for correlation

In studies which use DNA arrays, the expressions measured on an array will be correlated and these correlations imply that the set of p-values, {*p*_*i *_: *i *= 1,...,*m*}, are not independent. If the covariance of **z **is known, it is straightforward to modify the statistic so that it accommodates correlations. Suppose that cov(**z**) = **R**, then the variance of **1'z **is **1'R1 **and the appropriate p-value is



Note that the naïve estimator takes **R **= **I **implying no correlations among the *m *p-values.

When **R **is unknown, it must be estimated. In this context, it is useful to 'adjust' the variance of the naïve estimator. Let  be the average value of the off-diagonal elements of **R**, i.e.,  = (**1'R1 **- *m*)/(*m*(*m *- 1)), then the implied adjustment is



That is,



The correction for Ponly depends on the average correlation, , and not on the individual correlations. We believe that this allows one to generate an acceptable estimate in small data sets even though the individual correlations will be poorly estimated.

A couple of insights can be drawn from Equation (1). Since the adjustment is less than 1 when  > 0; a significance level based upon the naïve version is too small. Further, the need for adjustment increases with the size of the subset, i.e., *m*.

### Estimating a correction for correlation

Continuing with the data model described in the Introduction, the mean of *n *observations is assumed to have a multivariate normal distribution with mean vector, **Δ**, and covariance matrix, ; that is,



Let

• **S **estimate **Σ; **

• **D **be a diagonal matrix; ; estimated by 

The element-wise t-statistic for the null hypothesis, **Δ = 0**, can be written in vectorial form as



Since  is a consistent estimate of **D**, the t-test approximates the z-test for large *n*, i.e.,  as *n *increases. The cov(**z**) = **DΣD**, which is estimated by **S**.

Then for a one-sided *p*-value with an increasing alternative, **t → z **implies that the appropriate correlation matrix in Equation 1 is **R = DΣD**, which is approximated by  = **Σ**. Note that for a decreasing alternative, the same correlation applies for *z *= Φ^-1 ^(*p*).

### t-test

The one-sided p-value for a null hypothesis on **Δ **is based on the distribution of the statistic, . Let  and *u*_*j *_= **ay**_*j*_, and note that



Further, the variance of  satisfies



So



This provides an alternative way to compute Equation (1), which has some advantages. First, one only needs to know , which is computed in the by-gene analyses. Second, the by-gene analysis applied to {*u*_*j *_: *j *= 1,...,*n*} computes the statistic and its p-value directly. So algorithms are simpler. Finally, the p-value is based on a t-distribution, which better reflects the effect of small samples.

Morrison [19] derives Hotelling's *T*^2 ^test in the context of t-statistics of linear combinations, and presumably such statistics have a history nearly as long as multivariate statistics. O'Brien [20] examined this specific statistic as a method for comparing multiple endpoints in clinical trials. Lauter [21] noted that the null distribution is not the t-distribution with small sample sizes and explained a modified statistic, which corrects this. While the modified statistic controls the Type 1 error, the modification seems to reduce the power relative to O'Brien's statistic (simulations not reported).

### One- versus two-sided tests

So far, we have been discussing the case where *p*_i _is a one-sided *p*-value. Essentially, Equation 1 applies whether *p*_*i *_is a *p*-value from a one-sided test or a two-sided test. However, the covariance of **z **is not the same as the covariance of its absolute value, |**z**|. Consequently a two-sided test in which , needs a different adjustment than a one-sided test in which *p *= 1 - Φ(*z*).

For a two-sided test, the null distribution of **1'**|**z**| can be generated through Monte Carlo samples from the null distribution of **z**, MVN(**0**, cov(**z**)). That is, let **z**_1_,...,**z**_*k *_be pseudo-random samples from the multivariate normal distribution, MVN(**0**, cov(**z**)), and directly compute the *p*-value for the observed value, ψ = **1'**|**z**|, as



where I(*A*) is an indicator function which gives 1 if *A *is true, or 0 otherwise. In simulations where **Σ **is specified, the adjustment  can be implemented based upon **R**,  or **I**. In the two-sided case, it is also of interest to generate samples from MVN(**0**, cov(**z**)) where  is computed from  and . In theory, adjustments based upon **R **correct the p-value for correlations, and for large enough *n *so will  and . In practice,  or  must be useful when *n *is quite small. Their utility in this regard can be illustrated with simulated data.

## Results

### Simulation

The theory outlined in the previous sections provides adjustments which will work well for large sample sizes. It does not guarantee much when sample sizes are as small as is seen in most studies that use DNA arrays. We simulated a few 'representative' cases to illustrate that *P *computed from the naïve statistic can be very inaccurate and to demonstrate that the adjustments proposed herein give useful corrections with small sample sizes.

The 'representative' simulations assumed a covariance matrix, **Σ**, for *m *= 20 correlated genes. We constructed **Σ **= **D**^-1^**RD**^-1 ^by specifying **R **and **D**. The correlation matrix, **R**, is given in Table 1,  ≈ 0.45. These correlations were randomly selected to be between 0.35 and 0.55. Correlations in this range are commonly observed in our studies. Likewise, we randomly selected 20 variances in the range typical of our studies (Table 2). For a given sample size, *n*, pseudo-random samples **y**_1_,**y**_2_,...,**y**_*n *_were generated from a multivariate normal distribution, MVN(**0,Σ**), and *P *was computed using Equations 1 or 2. Since **Σ **is known, these computations can be based upon **R**, ,  or **I**. This procedure was iterated at least 10,000 times to observe enough *P*-values to adequately estimate the empirical distribution.

**Figure 1:**
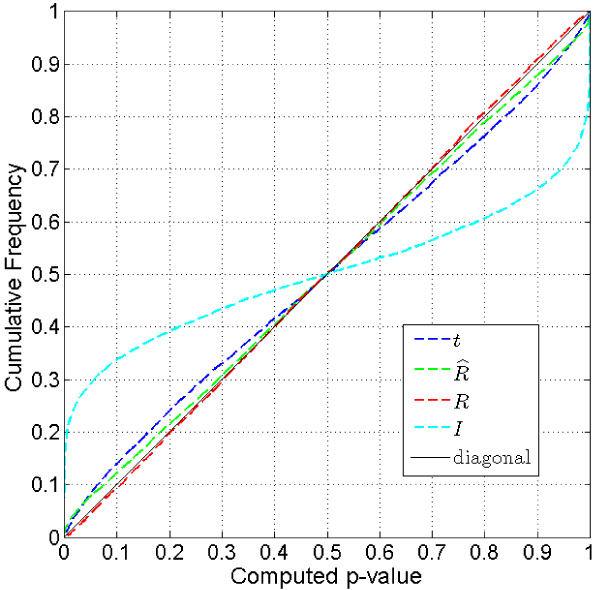
**Cumulative distribution of p-values for one-sided test case with sample size n = 5**. The corrected p-values based on a t-distribution (blue line) and the  (green line) are near diagonal, while the naïve p-value (cyan line) overstates the significance of the test. We can correct the problem when we know the true correlation, **R **between the genes in a GO term (red line).

Figure 1 plots the cumulative distributions of *P*-values from a one-side test with *n *= 5. Ideally the null p-values follow a uniform distribution, the diagonal line in this figure. The naïve *P*-value (cyan line) grossly overstates the significance of the test statistic with roughly 30% of these *P*-values being less than 0.05. The *P*-values (red line) computed using Equation 1 with the true correlation, **R**, have the expected distribution; the observed departures from the diagonal are within the variation associated with estimating the uniform distribution by an empirical distribution (Kolmogorov-Smirnov test, p = 0.21). The corrected p-values based on the t-test method (blue line) and the estimated correlation,  (green line), are near the diagonal albeit not perfect. However, they offer a big improvement over naïve values.

**Figure 2:**
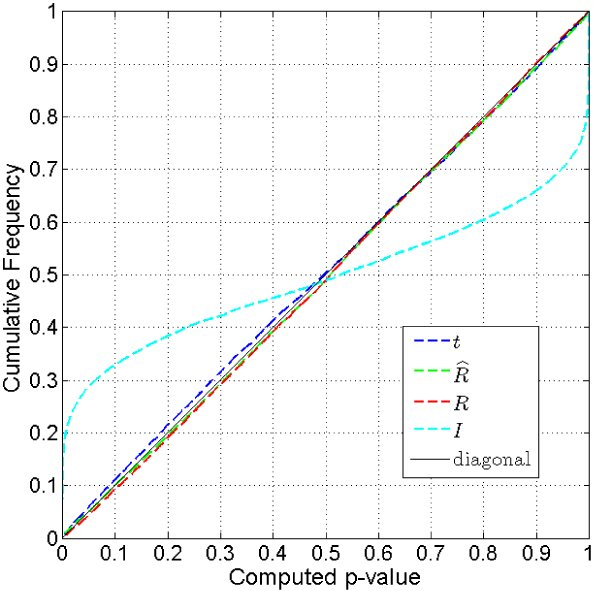
**Cumulative distribution of p-values for one-sided test case with sample size n = 15**. The corrections become better with a larger sample size. All of the correction methods give right adjustment as the prediction of the correlation becomes fairly accurate.

The corrections are expected to improve with larger sample sizes, and this is illustrated for *n *= 15 in Figure 2 where all of the correction methods are fairly accurate. Note also that the distribution for the naïve *P*-value does not respond to increasing sample size and remains very inaccurate. Figures 1 and 2 were generated from 10,000 iterations. Essentially, the empirical distributions for the corrections plotted in Figure 2 are not statistically different, and discrepancies with our expectations most likely reflect the variability among the plotted distributions. For example, we would expect the t-test version to be at least as good as the correction based on , and this is not apparent in Figure 2.

**Figure 3:**
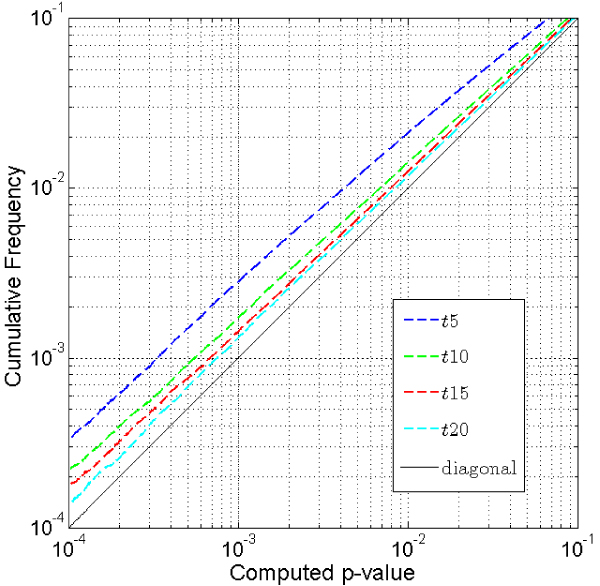
**Cumulative distribution of p-values for t-test correction with sample size n = 5, 10, 15, 20**. The convergence toward the diagonal as sample sizes increase is illustrated by the p-values in the interval, 0.0001 to 0.1, for the t-test correction where the empirical distributions were estimated from 1 million iterations.

In practice we use the t-test correction because it is easiest to compute. Figure 3 plots the interval, 0.0001 to 0.1, for the t-test correction where the empirical distributions were estimated from 1 million iterations. This figure illustrates the convergence toward the diagonal as sample sizes increase. Even at *n *= 5, the accuracy is acceptable for our purposes and represents a substantial improvement over the naïve calculation. For example, the nominal level of 0.05 would actually be 0.08; much closer to the nominal level than the naïve test at approximately 0.3 (Figure 1).

**Figure 4:**
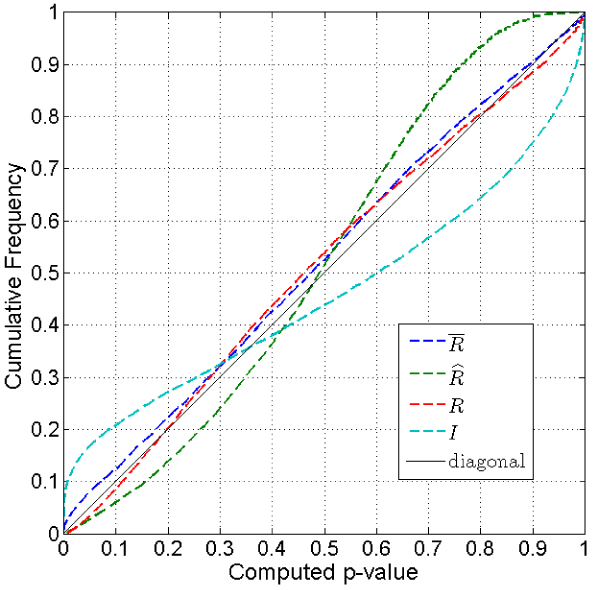
**Cumulative distribution of p-values for two-sided test case with sample size n = 5**. P-values calculated from random samples based on  (blue line) gives a reliable correction, which is comparable with the p-values from the true correlation **R **(red line).

**Figure 5:**
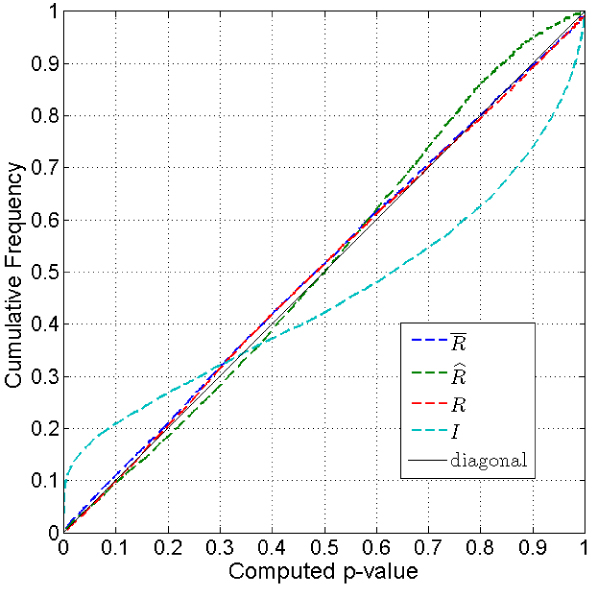
**Cumulative distribution of p-values for two-sided test case with sample size n = 15**. A better correction is also seen with larger sample sizes in two-sided case. In this case we have more accurate estimate of the true correlation **R**.

Figure 4 and Figure 5 show the results for simulations of the two-sided case, *n *= 5, 15 respectively. Clearly, the naïve *P*-values should not be used because they are inaccurate. The empirical distributions for the other methods in these figures involve considerable computation since each *P*-value is generated by Monte Carlo sampling. To speed up computations, we terminated Monte Carlo sampling when  or *k *= 10^6^. Consequently, small values of *P *are more accurately estimated than large values. In practice, we do not need to estimate large values with as much accuracy as small values so this is not a bad strategy. However, it makes it more difficult to evaluate whether the correction follows the diagonal. For small values, say < 0.1, the corrections are comparable in accuracy to those in the one-sided simulations. The correction based upon  (blue line) seems preferable since it appears to be more consistent with the correction based upon the true correlation, **R **(red line).

## Discussion

Correlations among gene expressions within a GO term invalidate the computed p-value when it is based upon assumed independence. Such estimates overstate the significance when the correlations are positive. This behavior was demonstrated analytically through a specific statistic, Equation (1), as well as through simulations. The simulated data show that the bias of the naïve p-value can be substantial with moderate correlation.

For didactic reasons, we used a statistic and a scenario, which is mathematically tractable. However, it should be understood that overstating statistical significance is a problem for any statistic where the computed p-value for the GO term assumes independence among gene expressions. This is true for widely implemented tests which evaluate if significant genes are 'over-represented' within a GO term. In these tests, p-values are based upon the hypergeometric distribution (Fisher's Exact Test) or its binomial or chi-squared approximations, and an assumption of independence is essential to the construction of the null distribution. In addition to the presented statistic, there are other meta-analysis tests based upon a uniform distribution of null p-values. A theory-based adjustment for correlation is difficult to construct for these tests. Naïve versions are biased, but not always as severe as the estimate presented here. So, we have been pursuing corrections for them.

For the illustrated statistic, the naïve p-value is easy to correct when the correlation is known. In practice the correlation must be estimated from limited data. Under the one-sample t-test scenario, we can estimate the applicable correlation. The simulation of a 'representative' group of 20 genes shows that estimating the correlation improves upon the naïve p-value with as few as 5 samples.

In the one-sided case, a t-statistic can be computed which implicitly adjusts for the presence of correlations. This statistic is easy to implement in existing computer programs; essentially the program that computed by-gene p-values can be used. This procedure can be extended to any statistical test that can be applied to the individual genes. As presented here, the one-sided alternative specifies that all genes change in the same direction. It is trivial to apply the procedure for any pre-specified direction of change for each gene. As our knowledge of expression profiles from responses to toxicity grows, this approach might become a standard test in screening chemicals for toxicity.

In an exploratory context, it is not possible to pre-specify how individual genes will respond to treatment, and p-values must reflect the two-sided alternative. At least with the simulated data, the  method worked well to control the size of the test. Because  misrepresents the true correlation structure, we are cautious in recommending its general use and plan to simulate a broader set of scenarios.

## Conclusion

Reliable corrections for the effect of correlations among genes on the significance level of a GO term can be constructed for a one-sided alternative hypothesis. For general two-sided alternatives the bias of naïve significance calculations can be greatly decreased although not eliminated.

## Authors' contributions

RD conceived of the ideas presented herein, developed the main methodology and wrote the manuscript. TL conducted the simulations. CV reviewed literatures. TL and CV have been involved in development of the algorithm and drafting the manuscript. All authors read and approved the final manuscript.

## Figures and Tables

**Table 1:** Correlation matrix, **R**, used in the simulations. The representative correlations were randomly selected to be between 0.35 and 0.55. These correlations in this range are commonly observed in our studies.

1	0.3722	0.412	0.3719	0.5174	0.3699	0.478	0.4798	0.4496	0.5085	0.5058	0.3705	0.3524	0.4731	0.4538	0.4057	0.4246	0.443	0.3885	0.4519
0.3722	1	0.3527	0.4034	0.5445	0.4073	0.522	0.5225	0.5097	0.5239	0.3733	0.5298	0.5443	0.3895	0.4547	0.5036	0.3541	0.5071	0.429	0.4089
0.412	0.3527	1	0.5456	0.359	0.5349	0.398	0.4338	0.3809	0.4903	0.409	0.3718	0.3743	0.5229	0.3965	0.5091	0.5334	0.413	0.3691	0.428
0.3719	0.4034	0.5456	1	0.5345	0.514	0.409	0.4894	0.5176	0.4007	0.3824	0.4371	0.3679	0.4261	0.4772	0.3796	0.3906	0.4217	0.4393	0.4452
0.5174	0.5445	0.359	0.5345	1	0.4061	0.418	0.4092	0.5346	0.5224	0.4241	0.3802	0.3807	0.5035	0.4824	0.4953	0.4104	0.3501	0.5438	0.5125
0.3699	0.4073	0.5349	0.514	0.4061	1	0.351	0.5327	0.434	0.4566	0.4948	0.4281	0.4867	0.4563	0.4714	0.5291	0.4009	0.354	0.503	0.4166
0.4776	0.5225	0.3979	0.4092	0.4176	0.3505	1	0.4777	0.4354	0.4761	0.4109	0.3624	0.5064	0.4444	0.4038	0.3903	0.4479	0.407	0.5217	0.5469
0.4798	0.5225	0.4338	0.4894	0.4092	0.5327	0.478	1	0.3583	0.444	0.4546	0.4606	0.4706	0.4674	0.4813	0.4581	0.5171	0.4625	0.4332	0.3834
0.4496	0.5097	0.3809	0.5176	0.5346	0.434	0.435	0.3583	1	0.5184	0.548	0.4173	0.5257	0.4726	0.3544	0.4549	0.4734	0.5458	0.4346	0.5143
0.5085	0.5239	0.4903	0.4007	0.5224	0.4566	0.476	0.444	0.5184	1	0.5253	0.5161	0.4758	0.3904	0.4799	0.4291	0.5031	0.473	0.3508	0.4288
0.5058	0.3733	0.409	0.3824	0.4241	0.4948	0.411	0.4546	0.548	0.5253	1	0.4822	0.4519	0.4779	0.4359	0.3542	0.4875	0.4398	0.3992	0.4385
0.3705	0.5298	0.3718	0.4371	0.3802	0.4281	0.362	0.4606	0.4173	0.5161	0.4822	1	0.5422	0.4317	0.4011	0.5234	0.4553	0.4305	0.432	0.391
0.3524	0.5443	0.3743	0.3679	0.3807	0.4867	0.506	0.4706	0.5257	0.4758	0.4519	0.5422	1	0.3732	0.352	0.4682	0.4535	0.3799	0.3696	0.5219
0.4731	0.3895	0.5229	0.4261	0.5035	0.4563	0.444	0.4674	0.4726	0.3904	0.4779	0.4317	0.3732	1	0.4225	0.3962	0.478	0.4375	0.5188	0.4279
0.4538	0.4547	0.3965	0.4772	0.4824	0.4714	0.404	0.4813	0.3544	0.4799	0.4359	0.4011	0.352	0.4225	1	0.4796	0.3642	0.5126	0.364	0.3575
0.4057	0.5036	0.5091	0.3796	0.4953	0.5291	0.39	0.4581	0.4549	0.4291	0.3542	0.5234	0.4682	0.3962	0.4796	1	0.41	0.5454	0.4097	0.3597
0.4246	0.3541	0.5334	0.3906	0.4104	0.4009	0.448	0.5171	0.4734	0.5031	0.4875	0.4553	0.4535	0.478	0.3642	0.41	1	0.3866	0.4484	0.5465
0.443	0.5071	0.413	0.4217	0.3501	0.354	0.407	0.4625	0.5458	0.473	0.4398	0.4305	0.3799	0.4375	0.5126	0.5454	0.3866	1	0.4062	0.3617
0.3885	0.429	0.3691	0.4393	0.5438	0.503	0.522	0.4332	0.4346	0.3508	0.3992	0.432	0.3696	0.5188	0.364	0.4097	0.4484	0.4062	1	0.3759
0.4519	0.4089	0.428	0.4452	0.5125	0.4166	0.547	0.3834	0.5143	0.4288	0.4385	0.391	0.5219	0.4279	0.3575	0.3597	0.5465	0.3617	0.3759	1

**Table 2:** Variances used in the simulations. 20 variances are randomly selected in the range typical of our studies.

0.058864
0.034697
0.025862
0.023389
0.002802
0.00839
0.000179
0.003993
0.014377
0.002584
0.000641
0.000972
0.003201
0.047617
0.051366
0.003047
0.004786
0.000368
0.007916
0.010357

## References

[B1] Tong W, Harris S, Cao X, Fang H, Shi L, Sun H, Fuscoe J, Harris A, Hong H, Xie Q, Perkins R, Casciano D (2004). Development of public toxicogenomics software for microarray data management and analysis. Mutat Res.

[B2] Khatri P, Draghici S (2005). Ontological analysis of gene expression data: current tools, limitations, and open problems. Bioinformatics.

[B3] Draghici S, Khatri P, Martins RP, Ostermeier GC, Krawetz SA (2003). Global functional profiling of gene expression. Genomics.

[B4] Johnson NL, Kotz S (1969). Discrete Distributions.

[B5] Allison DB, Gadbury GL, Heo M, Fernandez JR, Lee C-K, Prolla TA, Weindruch R (2002). A mixture model approach for the analysis of microarray gene expression data. Computational Statistics and Data Analysis.

[B6] Delongchamp RR, Bowyer JF, Chen J, Kodell RL (2004). Multiple-testing strategy for analyzing cDNA array data on gene expression. Biometrics.

[B7] Schweder T, Spjotvoll E (1982). Plots of p-values to evaluate many tests simultaneously. Biometrika.

[B8] Hedges LV, Olkin I (1985). Statistical Method for Meta-Analysis.

[B9] Brown MB (1975). A method for combining non-independent, one-sided tests of significance. Biometrics.

[B10] Kost JT, McDermott MP (2002). Combining dependent p-values. Statistics & Probability Letters.

[B11] Xu X, Tian L, Wei LJ (2003). Combining dependent tests for linkage or association across multiple phenotypic traits. Biostatistics.

[B12] Zaykin DV, Zhivotovsky LA, Westfall PH, Weir BS (2002). Truncated product method for combining *p *-values. Genetic Epidemiology.

[B13] Mootha VK, Lindgren CM, Eriksson KF, Subramanian A, Sihag S, Lehar J, Puigserver P, Carlsson E, Ridderstrale M, Laurila E, Houstis N, Daly MJ, Patterson N, Mesirov JP, Golub TR, Tamayo P, Spiegelman B, Lander ES, Hirschhorn JN, Altshuler D, Groop LC (2003). PGC-1alpha-responsive genes involved in oxidative phosphorylation are coordinately downregulated in human diabetes. Nat Genet.

[B14] Tian L, Greenberg SA, Kong SW, Altschuler J, Kohane IS, Park PJ (2005). Discovering statistically significant pathways in expression profiling studies. PNAS.

[B15] Simon R, Lam AP (2005). BRB Array-Tools User's Guide, Version 33: (National Cancer Institute Biometric Research Branch, Bethesda, MD) Technical Report.

[B16] Delongchamp RR, Velasco C, Dial S, Harris AJ (2005). Genome-wide estimation of gender differences in the gene expression of human livers: statistical design and analysis. BMC Bioinformatics.

[B17] Desai VG, Moland CL, Branham WS, Delongchamp RR, Fang H, Duffy PH, Peterson CA, Beggs ML, Fuscoe JC (2004). Changes in expression level of genes as a function of time of day in the liver of rats. Mutation Research – Fundamental & Molecular Mechanisms of Mutagenesis.

[B18] Parrish RS, Delongchamp RR, Allison DB, Page GP, Beasley TM, Edwards JW (2005). Normalization. DNA Microarrays and Statistical Genomic Techniques: Design, Analysis, and Interpretation of Experiments.

[B19] Morrison DF (1967). Multivariate Statistical Methods.

[B20] O'Brien PC (1984). Procedures for comparing samples with multiple endpoints. Biometrics.

[B21] Lauter J (1996). Exact t and F tests for analyzing studies with multiple endpoints. Biometrics.

